# Compact dual-mode diffuse optical system for blood perfusion monitoring in a porcine model of microvascular tissue flaps

**DOI:** 10.1117/1.JBO.22.12.121609

**Published:** 2017-12-14

**Authors:** Seung Yup Lee, Julia M. Pakela, Michael C. Helton, Karthik Vishwanath, Yooree G. Chung, Noah J. Kolodziejski, Christopher J. Stapels, Daniel R. McAdams, Daniel E. Fernandez, James F. Christian, Jameson O’Reilly, Dana Farkas, Brent B. Ward, Stephen E. Feinberg, Mary-Ann Mycek

**Affiliations:** a University of Michigan, Department of Biomedical Engineering, Ann Arbor, Michigan, United States; b University of Michigan, Applied Physics Program, Ann Arbor, Michigan, United States; c Miami University, Department of Physics, Oxford, Ohio, United States; d Radiation Monitoring Devices Inc., Watertown, Massachusetts, United States; e Northeastern University, Boston, Massachusetts, United States; f University of Michigan, Department of Oral and Maxillofacial Surgery, Ann Arbor, Michigan, United States

**Keywords:** diffuse correlation spectroscopy, diffuse reflectance spectroscopy, blood flow, blood perfusion, porcine model, free tissue flap

## Abstract

In reconstructive surgery, the ability to detect blood flow interruptions to grafted tissue represents a critical step in preventing postsurgical complications. We have developed and pilot tested a compact, fiber-based device that combines two complimentary modalities—diffuse correlation spectroscopy (DCS) and diffuse reflectance spectroscopy—to quantitatively monitor blood perfusion. We present a proof-of-concept study on an *in vivo* porcine model (n=8). With a controllable arterial blood flow supply, occlusion studies (n=4) were performed on surgically isolated free flaps while the device simultaneously monitored blood flow through the supplying artery as well as flap perfusion from three orientations: the distal side of the flap and two transdermal channels. Further studies featuring long-term monitoring, arterial failure simulations, and venous failure simulations were performed on flaps that had undergone an anastomosis procedure (n=4). Additionally, benchtop verification of the DCS system was performed on liquid flow phantoms. Data revealed relationships between diffuse optical measures and state of occlusion as well as the ability to detect arterial and venous compromise. The compact construction of the device, along with its noninvasive and quantitative nature, would make this technology suitable for clinical translation.

## 1 Introduction

In reconstructive surgery, tissue perfusion/vessel patency is critical to the success of microvascular-free tissue flaps. In the case of impending failure due to compromise in vascular perfusion of tissue, prompt surgical intervention following early detection is correlated with the flap salvage rate. About 5% to 25% of free flaps necessitate re-exploration due to circulatory/vascular compromise, with a salvage rate reported as 44.9%.^1^ The majority of microvascular thrombi occur within the first 24 to 48 postoperative hours. Thus, close and frequent monitoring during this time window is critical. Physical examination findings, such as flap color, temperature, and capillary refill/perfusion rate, are currently considered gold-standard techniques for postoperative microvascular-free flap monitoring. However, those methods require trained personnel who make infrequent visits for hands-on evaluations; this can delay the identification of perfusion issues. In addition, these personnel rely on subjective assessments, possibly leading to observer variability.

For continuous and objective monitoring, perfusion-monitoring devices—including color duplex ultrasonography, implantable ultrasound Doppler, laser Doppler flowmetry (LDF), and near-infrared visible light spectroscopy—are currently employed in clinics. Although they provide continuous information about perfusion, imaging devices still require an expert’s interpretation, the implantable ultrasonic Doppler is invasive,^2^ and LDF is known to be sensitive to a superficial layer.^3^^,^^4^ Recently, multimodal optical spectroscopy (reflectance + autofluorescence spectroscopy)^5^ and an advanced quantitative optical imaging system^6^ have been employed to characterize tissue flap perfusion, but the former does not evaluate blood flow directly and the latter requires complex instruments in spite of providing spatial information.

Here, we present a compact, multimodal optical spectroscopy system to provide noninvasive, accurate, and continuous monitoring of complimentary perfusion parameters: microvascular blood flow information, tissue oxygenation, and hemoglobin concentration. The system consists of two diffuse optical techniques: diffuse correlation spectroscopy (DCS) and diffuse reflectance spectroscopy (DRS). As a relatively new optical technique to measure blood flow in deep tissues, DCS measures the temporal intensity fluctuation of speckle patterns formed by scattering moving particles, such as red blood cells in blood vessels. The intensity fluctuation’s temporal autocorrelation is related to blood flow information through the diffusion equation (DE) of light propagation inside turbid media.^7^^,^^8^ DCS has been validated against different flow-measuring techniques^9^^,^^10^ and employed in a variety of preclinical and clinical settings.^11^^,^^12^ DRS is a well-established technique to noninvasively estimate tissue oxygenation and total hemoglobin concentration when it is coupled with tissue-optics models analyzing the tissue reflectance spectra. Due to its simple configuration but powerful performance, DRS has been substantially employed in a variety of clinical and preclinical applications.^13^^–^^17^ Since previous studies have reported that technologies that solely monitor blood flow have failed to distinguish between arterial and venous failure,^18^^,^^19^ we expect that the DRS-derived oxygenation and hemoglobin concentration supplementary to DCS measurements could help clinicians in identifying different types of flap failure.

Typically, DCS is constructed with a hardware correlator, a long-coherence laser, and a single-photon counter, which results in a relatively large footprint. However, this custom DCS system has been built using a software correlator and a smaller laser diode (LD), thus reducing the overall size to half that of the conventional DCS system. The DRS system also utilizes a state-of-the-art miniaturized spectrometer and high-power white light-emitting diode (LED), minimizing its footprint. The compactness of the device potentially allows for multichannel monitoring on different sites of interest in the flaps (both the external and buried flap) along with custom fiber-optic probes. To verify the performance of this diffuse-optic-based perfusion-monitoring device, we tested our DCS system in a flow phantom and employed an *in vivo* porcine flap model with a controllable arterial blood flow supply (n=4). For further validation and to demonstrate the utility of our device, we performed anastomosis on an additional four animals to simulate a free flap transfer procedure and conducted 5-h-long monitoring, followed by arterial and venous occlusion in case no spontaneous flap failure was found. A portion of the results was reported in Refs. 20 and 21. Here, we present findings from the expanded data set.

## 2 Methods

### 2.1 Instrumentation

**Fig. 1:**
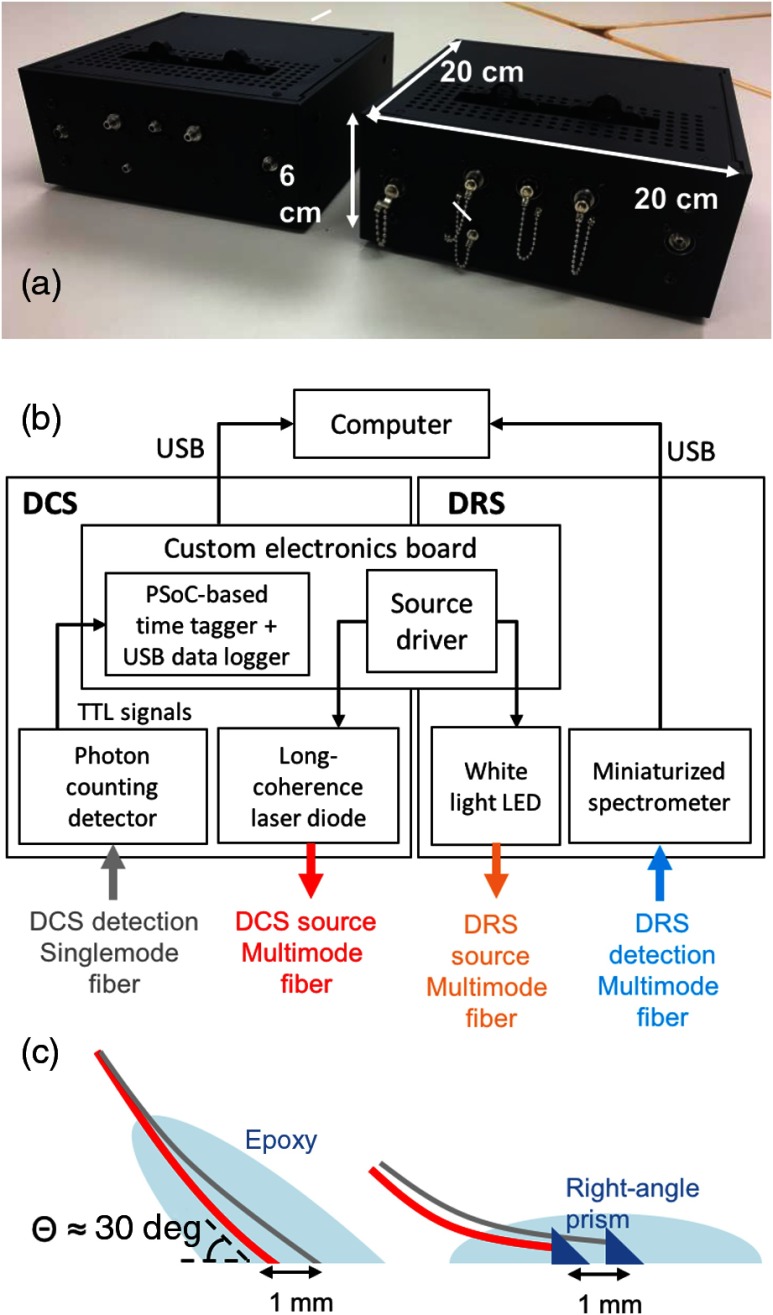
(a) Photo of the compact multimodal flap perfusion-monitoring device that evaluates complimentary perfusion parameters, including blood flow, tissue oxygenation, and hemoglobin concentration, by combining two-channel DCS and one-channel DRS—due to its compact footprint, multiple devices can be stacked in a constrained space for multisite flap monitoring. (b) Block diagram of the device shows the major components of each modality—the components have been carefully selected to achieve miniaturization while retaining comparable performance to the conventional DCS construction (long coherence LD, hardware correlator, and single-photon counting detector) and DRS (halogen lamp + intensified charged-couple device). (c) Implantable DCS probes are designed in two ways: using angle-polished fibers with final entry angle of 60 deg (left) and using a right-angle prim potted in an epoxy (right).

The instrument consisting of two-channel DCS and one-channel DRS was built in a small form factor (20  cm×20  cm×6  cm) as shown in Fig. 1(a). The construction details can be found in our previous reports.^22^^,^^23^ The core DCS components that enabled this miniaturization were a relatively inexpensive and small 785-nm LD (LD785-SH300, Thorlabs, Newton, New Jersey) and a custom, hardware-based time tagger using a programmable system-on-a-chip (PSoC) for implementing a software correlator. While conventional DCS systems employ stabilized ultralong coherence length (>100  m) lasers, we selected the current LD because the measured coherence length (∼4.5  m) exceeded plausible path-length distributions of detected photons for the given source–detector separations (<5  mm) in our application.^24^ A PSoC-based time tagger recorded each single photon’s arrival time with 80-ns resolution from transistor–transistor logic pulses generated by a single-photon counting module (SPCM-AQRH, Excelitas, Waltham, Massachusetts) commonly used in DCS. The time-stamped data were transferred via universal service bus (USB) (max rate=150  kHz/channel) to a remote computer that calculates autocorrelations using a multitau algorithm^25^^,^^26^ or saves raw data for other algorithms computing the autocorrelations, such as a fast Fourier transform (FFT) method.^27^ We validated this PSoC-based hardware coupled with multitau approach against a commercial hardware correlator, and the results showed that the difference in respective correlation functions was only at the jitter time scale in the PSoC-based tagger running at 12 MHz.^28^ Likewise, the miniaturized, visible, and near-infrared spectrometer (Avaspec-Mini, Avantes, The Netherlands) and high-power white LED (LCW W5SN, Osram, Sunnyvale, California) were employed to construct a DRS system with a minimal footprint. The additional electronics board controlled the intensities of the LD and LED light sources.

For light delivery and collection to and from different tissue flap sites, we employed different types of custom-made optical probes: a skin patch and two types of implantable probes (∼30-deg angle fiber and right-angle, prism-coupled fiber) [Fig. 1(c)]. The manufacturing process and testing results were reported in detail in Ref. 29. Multimode fibers (FG200LEA, Thorlabs) were used for the DCS source, DRS source, and DRS detection (core sizes were 200, 100, and 200  μm, respectively). A single-mode fiber with a core size of 4.4  μm (780HP, Thorlabs) was used for DCS single-photon detection. The angled fiber and the right-angled, prism-coupled fiber monitored the pedicles (artery in this case) directly or from the underside of a raised tissue flap—which mimics implanted monitoring in a buried flap—while the skin patch monitored the skin side of the flap to demonstrate a noninvasive evaluation of an exposed part of the flap.

Custom graphic user interface (GUI) software was developed in C# to provide multiplexed data acquisition between simultaneous four-channel DCS and one-channel DRS. The user easily selected the acquisition-related input parameters, such as LD and LED intensities, their integration times, and acquisition timing. Raw photon counts and autocorrelation plots were calculated via multitau methods and displayed in real time for four DCS channels along with a reflectance spectrum. Before this *in vivo* study, the basic performance of the entire system (device + optical probes + GUI) was verified on tissue-simulating phantoms.^30^^,^^31^

### 2.2 Flow Phantom Verification for DCS 

A liquid phantom was prepared using a 1.45  g/L mixture of titanium dioxide and silicone-based oil to mimic a scattering liquid. The oil was pumped through index matching silicone tubing (inner dia. = 1.5 mm × outer dia. = 3.5 mm) using a variable flow peristaltic pump (Fisher Scientific, CAT# 138764, Hampton, New Hampshire). Once the tubing was filled with oil, the pump was turned off to gather a baseline measurement simulating “no-flow.” The custom DCS system was used to take 100 measurements at a 5-s integration time for 2- and 5-mm source–detector separations. Four additional trials were taken at four different dial settings on the peristaltic pump to replicate varying flow speeds. Each trial followed the same procedure as no-flow by performing 100 measurements, and the average of these measurements was taken as the representative for each dial setting. Later, the same peristaltic pump was used to quantify the flow speeds of each dial setting by measuring the amount of time it took the pump to fill a beaker with 30 mL of silicone oil.

### 2.3 Animal Studies

**Fig. 2:**
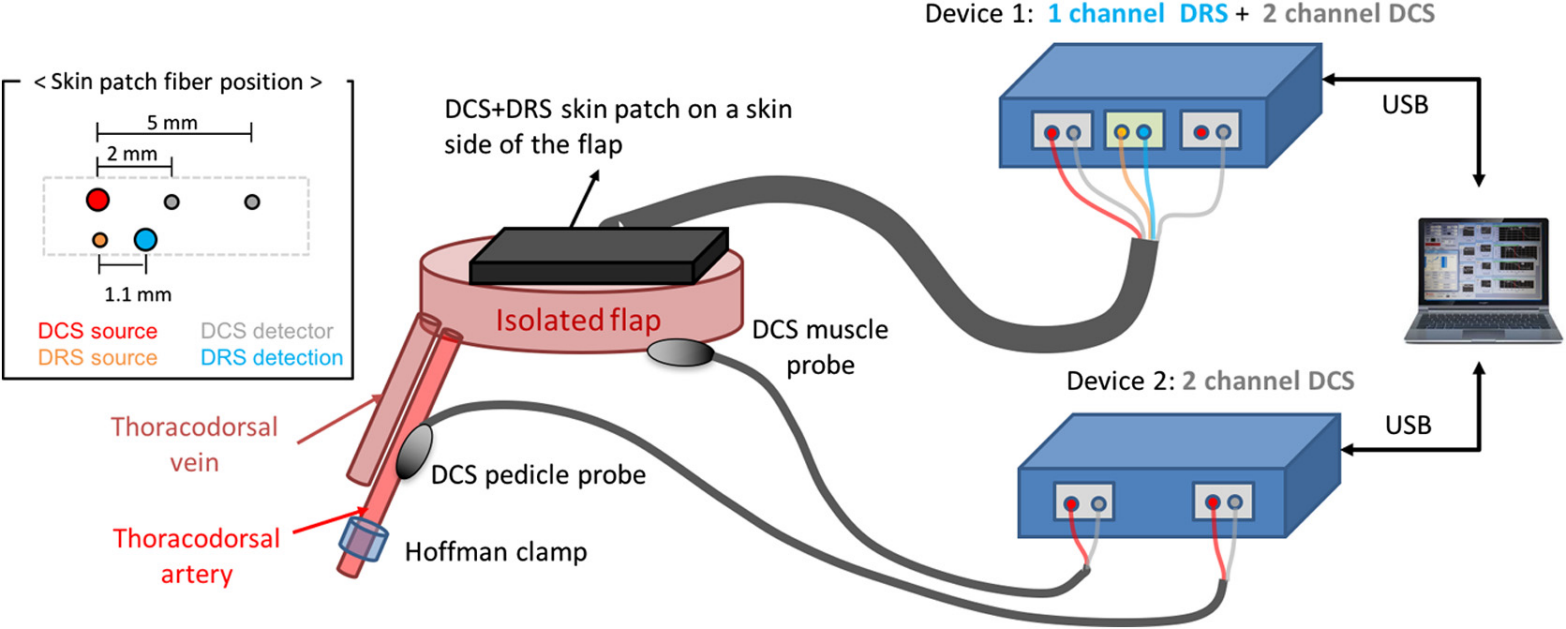
Two identical perfusion-monitoring devices (controlled by custom software in one laptop via USB) have been set up for multisite flap monitoring through different types of optical probes: skin patch, pedicle, and muscle probe. The inset diagram displays probe geometry for DCS and DRS in the skin optical patch.

**Fig. 3:**
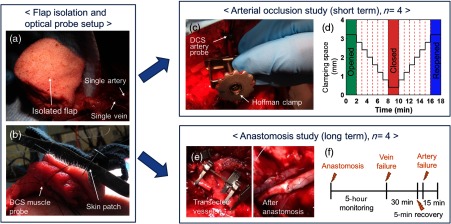
Animal study protocol. (a and b) After isolating the flap and securing the optical probes, we conducted two different studies on each of four animals: (1) (c and d) arterial occlusion study and (2) (e and f) anastomosis study. (a) The surgeon elevated a latissimus dorsi myocutaneous flap with a single artery and vein serving as the sole supply and drain channels for the flap. (b) The skin optical patch was fixed onto the skin side of the flap for one- and two-channel DCS measurements. The DCS muscle probe was sutured on the muscle side of the flap. (c) The Hoffman clamp incrementally occluded the supplying artery to simulate vascular compromise while sequential data acquisition of DCS and DRS was performed for all the flap sites. (d) The opening distance of the Hoffman clamp as a function of time, in minutes. (e) The vessels were transected (left) and reconnected (right) via anastomosis to simulate free flap transfer. (f) Protocol for long-term monitoring after anastomosis.

All animal experiments were performed in accordance with the University of Michigan Institutional Animal Care and Use Committee guidelines and approval. Figure 2 shows a instrumental setup for animal studies and Fig. 3 displays animal procedure in detail. Pigs of ∼15  kg were premedicated with telazol and xylazine on a per kg basis followed by a tracheostomy to establish a secure airway. Animals were maintained at 5% isoflurane general anesthesia and monitored for the experiment’s duration. A neck vein was accessed, and an intravenous channel was established. A latissimus dorsi myocutaneous flap was raised using standard surgical procedures. The arterial and venous supplies were traced back to a single artery and vein [Fig. 3(a)].

After the flap was isolated, optical probes were secured on three sites: the artery, the muscle side of the flap, and the skin side of the flap [Figs. 2, 3(b) and 3(c)]. The optical probe for skin monitoring had one source–detector pair for DRS (1.1-mm separation) and two DCS detector fibers paired to one DCS source fiber (separations were 2 and 5 mm, respectively). For the artery and muscle sites, a pedicle probe (one DCS source and detector pair with 1-mm separation) was employed (Fig. 2). Typically, the mean light penetration depth is on the order of half the source–detection separation distance in biological tissues.^32^ The DRS skin patch interrogates a very shallow depth (∼0.4  mm) underneath the skin surface to investigate perfusion in microvasculature. The DCS 2-mm probe forms an optical interrogation volume slightly larger than the DRS probe but is still sensitive to the shallower area, as compared to the DCS 5-mm probe’s deeper sensing area.

The gold standard for flow monitoring during these procedures was provided via direct observation from the surgical team. Specifically, surgeons looked for signs of discoloration and temperature changes to identify compromised blood perfusion. A venous compromise results in tissue acquiring a purple hue as deoxygenated blood pools in the flap, while an arterial compromise results in tissue acquiring a white hue as blood drains from the flap.

#### 2.3.1 Occlusion experiments

For the artery occlusion study, a Hoffman clamp positioned over the artery was varied such that a gap in the clamp was decreased from 3.2 mm (wide open) to 0.4 mm (full occlusion) by a step size of 0.4 mm every minute and then loosened back to 3.2 mm using the same step pattern [Fig. 3(d)]. For pigs 1 and 2, DCS and DRS data were measured during separate occlusion sequences. For pigs 3 and 4, DCS and DRS data were acquired together in rapid succession during the same occlusion sequence. Measurements for both modalities were taken on a 7-s cycle, with a signal length of 5 s and an integration time of 0.2 s for DCS and DRS, respectively, and a computing overhead time of 1.8 s. Each occlusion experiment lasted for ∼20  min.

#### 2.3.2 Anastomosis experiments

The free flap transfer involves reanastomosis of an artery and vein from a donor site to the vasculature of the recipient site vessels. After the flap was isolated with a single supplying artery and draining vein as described in Sec. 2.3, the artery and vein were transected and then anastomosed via standard microsurgical techniques [Fig. 3(e)]. Using the same probe configuration for DCS and DRS measurements, the flap was monitored for 5 h. When no spontaneous flap failure was observed by the physical exams during the 5-h monitoring period, the surgical team ligated the vein and then the artery with sutures to simulate venous and arterial failure, respectively, followed by 20- to 30-min and 5- to 15-min monitoring of each type of vascular compromise [Fig. 3(f)].

### 2.4 Diffuse Correlation Spectroscopy Analysis

Section 2.4.1 provides a detailed description of the analysis methods for the DCS results presented in this study. The methods used for this publication were chosen in order to create an analysis framework insensitive to the noise inherent to the data. For data analysis, we chose to utilize a quantitative parameter, $\tau_{1/2}$, which describes shape changes of the autocorrelation curve across scans and is independent of theoretical models. This choice was motivated by the desire to avoid additional uncertainties introduced by approximating the necessary optical coefficients for the DE.

#### 2.4.1 Calculation of autocorrelation function

As described in Sec. 2.1, the software saved time-stamped raw data for postprocessing. In addition, we calculated the intensity autocorrelation plot using a fast Fourier transform (FFT). For the data presented in this paper, we applied an FFT technique on raw data to generate autocorrelation functions from the distribution of photon arrival times and quantify temporal light intensity fluctuations.^33^ The intensity autocorrelation function [$g_{2}(\tau )$] was directly computed from the measured photon intensity using

$$g_{2}(\tau )=\frac{F^{-1}\{F[I(t)]\cdot F^{*}[I(t)]\}}{{\left\langle I(t)\right\rangle }^{2}},$$ (1)

where $F^{-1}$, F, and $F^{*}$ are the inverse FFT, the FFT, and its complex conjugate, respectively. I(t) is the intensity signal, defined as an indexed vector $I_{j}$, where bin number j represents a time interval during which a photon packet was collected. The resulting $g_{2}$ curves were then smoothed using a logarithmic filter.^33^ Specifically, the raw $g_{2}$ curves were downsampled across a logarithmically spaced set of τ values.

The data presented in this publication were acquired using the FFT method. As described in Sec. 2.1, the device software automatically calculates autocorrelation curves via a remote computer using the multitau method, which has been validated in previous studies.^28^ We implemented the FFT method to further validate this code; DCS analyses were conducted using $g_{2}$ functions from both the FFT and the multitau method (described in Sec. 2.1), and they were found to yield consistent results.

#### 2.4.2 Calculation of $\tau_{1/2}$

To monitor changes in blood flow, we computed the τ value corresponding to the point where the $g_{2}$ curve decayed halfway between its maximum and minimum values ($\tau_{1/2}$). This $\tau_{1/2}$ value was our estimate for relative blood flow.

Ideally, an autocorrelation curve displays well-defined plateaus before and after decay. The average values of the top (bottom) plateaus of the measured intensity autocorrelation mark the maximum (minimum) point on the curve used to define $\tau_{1/2}$. Some of the autocorrelation curves did not have well-defined top or bottom plateaus; thus, it was difficult to determine where the $g_{2}$ curve had decayed to halfway between its maximum and minimum value.

**Fig. 4:**
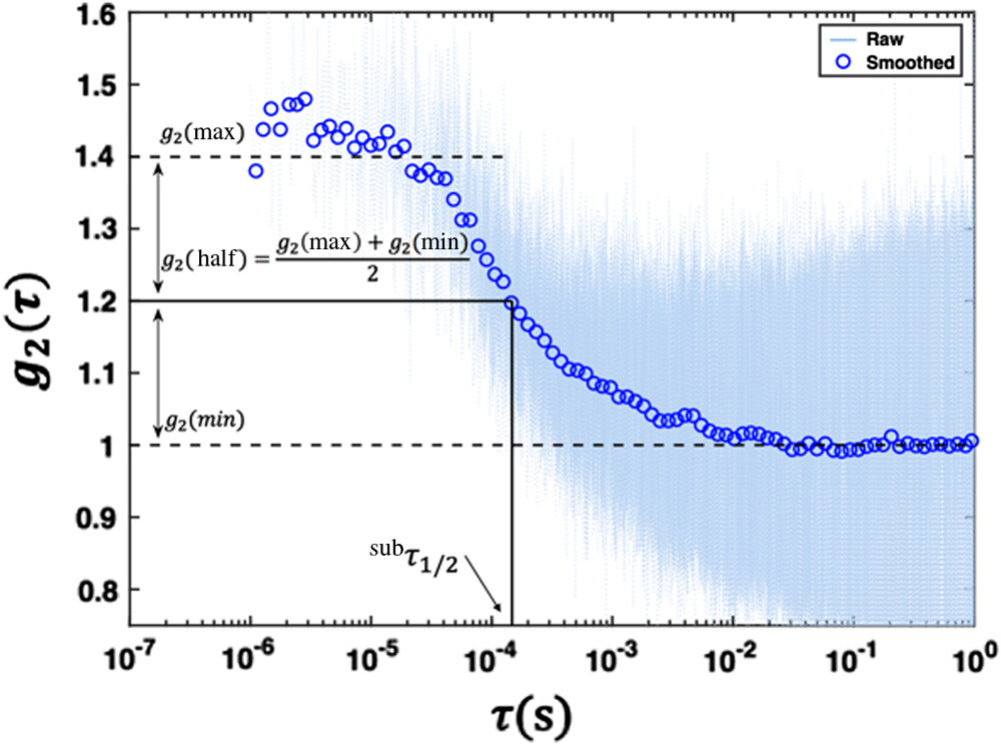
Representative curve showing the τ1/2sub calculation from a smoothed DCS curve superimposed over the raw data—smoothed curves were obtained by applying a logarithmic filter to the raw data. τ1/2sub values were calculated by finding the point where the autocorrelation ($g_{2}$) curve had decayed to halfway between its maximum and minimum value. Maximum (minimum) $g_{2}$ values were estimated by calculating the average $g_{2}$ value over a predefined range at the top (bottom) of the curve. A distribution of τ1/2sub values was obtained using different sets of feasible top and bottom ranges. A representative $\tau_{1/2}$ value for each scan was calculated as the average over the distribution of τ1/2sub values.

In order to mitigate situations in which the autocorrelation curves did not have well-defined plateaus, the method for calculating $\tau_{1/2}$ was designed to find representative $\tau_{1/2}$ values for each curve. For each $g_{2}$ curve, a set of many possible top and bottom plateau ranges were identified. The mean $g_{2}$ value from each of these potential plateau ranges was calculated. For every pair of potential minimum and maximum $g_{2}$ values, an intermediate $\tau_{1/2}$ value (τ1/2sub) was calculated as demonstrated in Fig. 4. The representative $\tau_{1/2}$ value for each scan was reported as the average across the distribution of τ1/2sub. This method was both computationally simple and effective at averaging over the noise associated with each curve.

$\tau_{1/2}$ values were used to quantify the measured change from baseline during the occlusion experiments. For the short-term studies, baseline was defined as the set of scans taken during the first and last 2 min of an experiment, when the Hoffman clamp was maximally opened. For the long-term studies, baseline was defined as the first three measurements taken during artery and venous failure simulations. For the DCS data, the measured response was calculated as the percent difference between the average $\tau_{1/2}$ values at baseline and maximum occlusion. The uncertainty in this measurement was calculated using the propagation of error equation as a function of the mean and standard deviations of the baseline and maximally occluded $\tau_{1/2}$ values.

### 2.5 Diffuse Reflectance Spectroscopy Analysis

**Fig. 5:**
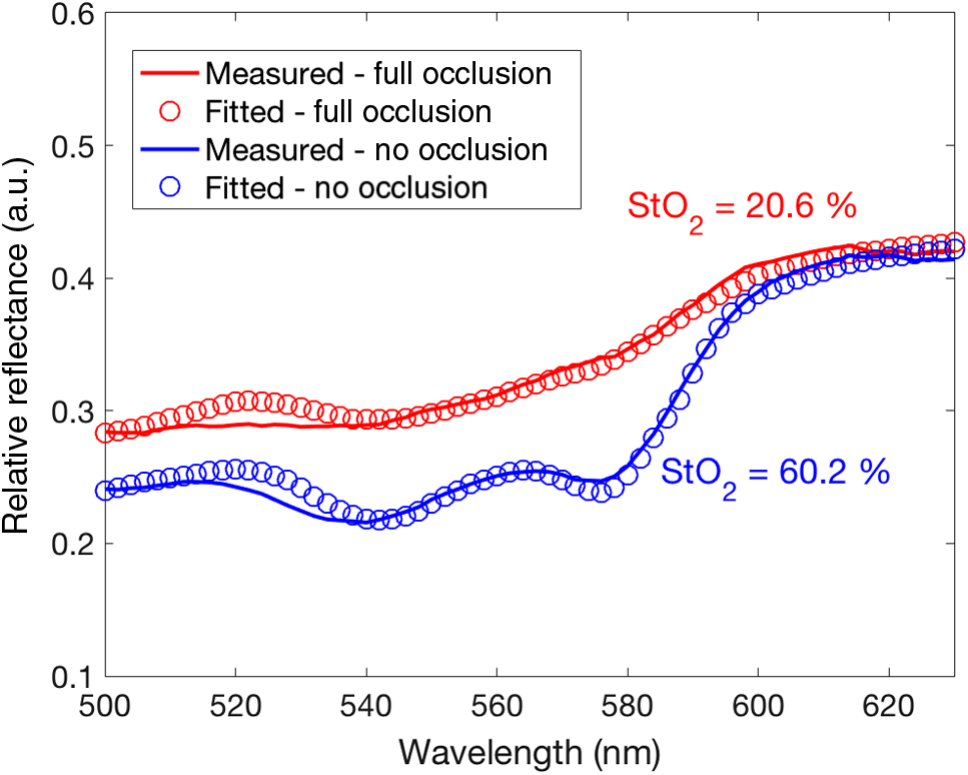
Representative reflectance spectra (solid lines) postprocessed (smoothed and corrected with the system response) at no occlusion (blue) and full occlusion (red) in pig 4 revealed a distinct difference in spectral shape. The model fits (hollow circles) based on an inverse algorithm quantified the oxygen saturation and total hemoglobin concentration.

The measured raw diffuse reflectance spectra were corrected with a system response obtained using a 50% Spectralon^®^ standard (SRS-50-010, Labsphere Inc., North Sutton, New Hampshire) and dark measurements collected without white light excitation. The corrected spectra were smoothed using a moving average filter. A Monte Carlo lookup table (MCLUT)-based inverse algorithm was applied on these smoothed, corrected reflectance spectra to extract two skin perfusion parameters: total hemoglobin concentration [HbT] and tissue oxygenation, ${\mathrm{StO}}_{2}$ (Fig. 5). The detailed principle of the MCLUT-based estimation is described in previous literature.^22^^,^^34^ Briefly, the model reflectance spectrum was created by the lookup table (LUT) constructed using Monte Carlo simulations run on scattering and absorption coefficients covering a range of biological tissue [Eq. (2)]. A wavelength-dependent reduced-scattering coefficient was described by a power law model, which is commonly accepted in tissue optics^35^ [Eq. (3): A is a scattering amplitude at a reference wavelength and B is a scattering power]. The absorption coefficient was expressed in a linear combination of oxy- and deoxyhemoglobin concentrations based on an assumption that hemoglobin is a major chromophore in the skin flap [Eq. (4): [HbT] is the total hemoglobin concentration, ${\mathrm{StO}}_{2}$ is the tissue oxygen saturation, $\epsilon_{\mathrm{HbO}}$ and $\epsilon_{\mathrm{Hb}}$ are the molar extinction of oxy- and deoxyhemoglobin, respectively]. The model reflectance was fitted to the measured reflectance in an iterative process to find the best parameters minimizing the percent errors between models and experiments using *fmincon*, a nonlinear, constrained optimization function available in the MATLAB^®^ optimization toolbox. A scaling ratio was applied to directly compare the model and experimental reflectance in the algorithm. The ratio was calculated by measuring a 1-μm-sized polystyrene microsphere (07310-15, Polysciences Inc., Warrington, Pennsylvania) mixture in deionized water with known optical properties computed by Mie theory.

The LUT was constructed using the embedded Monte Carlo functions in a ZEMAX^®^ nonsequential mode. The source and detection fibers were modeled with concentric cylinder objects with different refractive indices for core and cladding to make a numerical aperture (NA=0.22) of the used multimode fiber. A total of 252 simulations were run for the combinations of 21 increments of $\mu_{s}$ (0 to $40\;{\mathrm{cm}}^{-1}$) and 12 increments of $\mu_{a}$ (0.01, 0.5, 1 to $19\;{\mathrm{cm}}^{-1}$). A total of $10^{7}$ rays were launched for each run. The anisotropy, g, was 0.9, and the Henyey–Greenstein phase function was used for the scattering angle

$$R_{\text{model}}(\lambda )=f_{\mathrm{LUT}}[\mu_{a}(\lambda ),\mu_{s}^{\prime }(\lambda )],$$ (2)

$$\mu_{s}^{\prime (\lambda )}=A{\left(\frac{\lambda }{\lambda_{o}}\right)}^{-B},$$ (3)

$$\mu_{a}(\lambda )=\ln(10)\times [\mathrm{HbT}]\times [{\mathrm{StO}}_{2}\times \epsilon_{\mathrm{HbO}}+(1-{\mathrm{StO}}_{2})\times \epsilon_{\mathrm{Hb}}].$$ (4)

[HbT] and ${\mathrm{StO}}_{2}$ were used to quantify the measured change from baseline during the occlusion experiments. Baseline is defined as the set of spectra measured during the first and last 2 min of an experiment, when the Hoffman clamp was fully opened.

For the DRS data, measured response was calculated for both [HbT] and ${\mathrm{StO}}_{2}$ as the percent difference between the values extracted from baseline spectra and those extracted from maximum occlusion. The value for baseline and uncertainty for the short- and long-term study were calculated using the same method as the DCS data described in Sec. 2.4.2.

## 3 Results

### 3.1 Phantom Studies

**Fig. 6:**
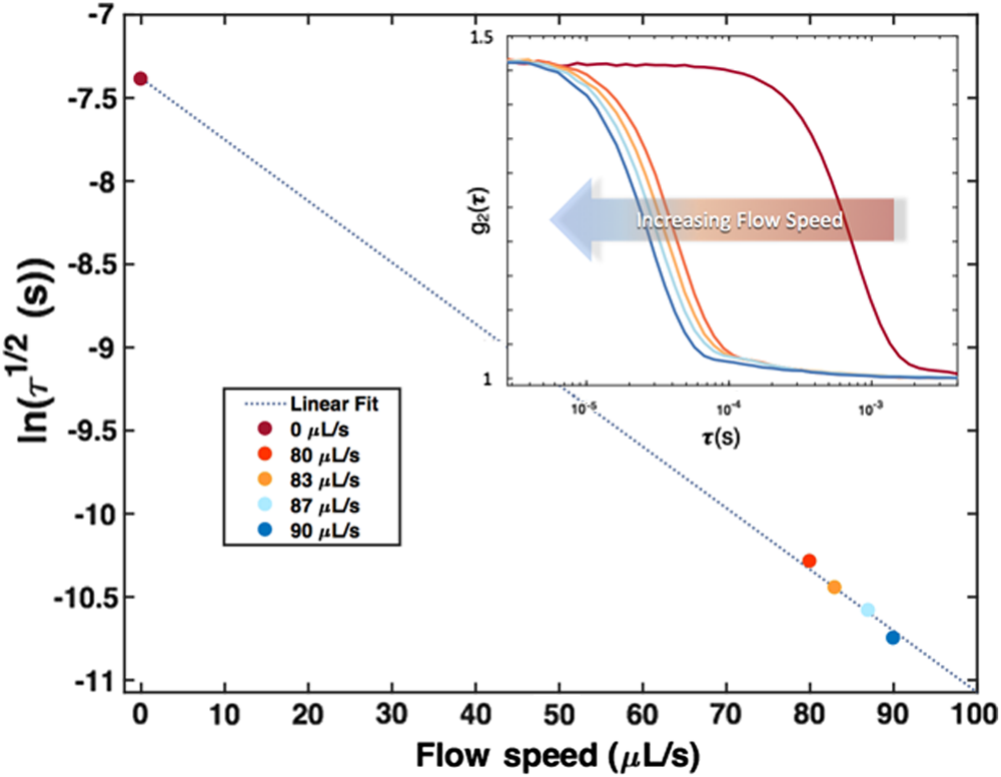
An average $\tau_{1/2}$ value was calculated for five different flow speeds from the 100 scans taken at each dial setting. The log of the average $\tau_{1/2}$ value was plotted against its corresponding flow speed as listed in the legend. The uncertainty associated with the $\tau_{1/2}$ values ranged between 5% and 6%. A linear model was fitted (dashed line) from the five data points that show a linear relation ($R^{2}=0.99$) between the flow speed and $\log(\tau_{1/2})$. The subplot of the average autocorrelation curves for five different flow speeds further show this relation qualitatively. An immediate leftward shift in the autocorrelation curve is observed when moving from a no-flow state (maroon curve) to a flow state (red, orange, and blue curves). A gradual shift is observed when increasing the speed of the flow state. These results indicate that the device can differentiate between a flow and no-flow state with some sensitivity to changing flow rates.

Figure 6 summarizes the results of the flow-phantom studies described in Sec. 2.2. The logarithm of the averaged $\tau_{1/2}$ value at each dial setting is plotted against the corresponding flow speed. The uncertainty associated with the $\tau_{1/2}$ values ranged between 5% and 6%. A linear fit ($R^{2}=0.99$) was then calculated and plotted (dashed line) using the five data points from each dial. The subplot shows the average autocorrelation curves for each dial setting. A large leftward shift in the autocorrelation curve was observed when going from a no-flow state to a “flow” state. A gradual shift toward the left was then observed as the flow rate was increased. This is further exhibited by the strong linear relation between the flow speed and $\log(\tau_{1/2})$. All data presented are for a source–detector separation of 2 mm. A source–detector separation of 5 mm was also analyzed and yielded comparable results.

### 3.2 Occlusion Studies

**Fig. 7:**
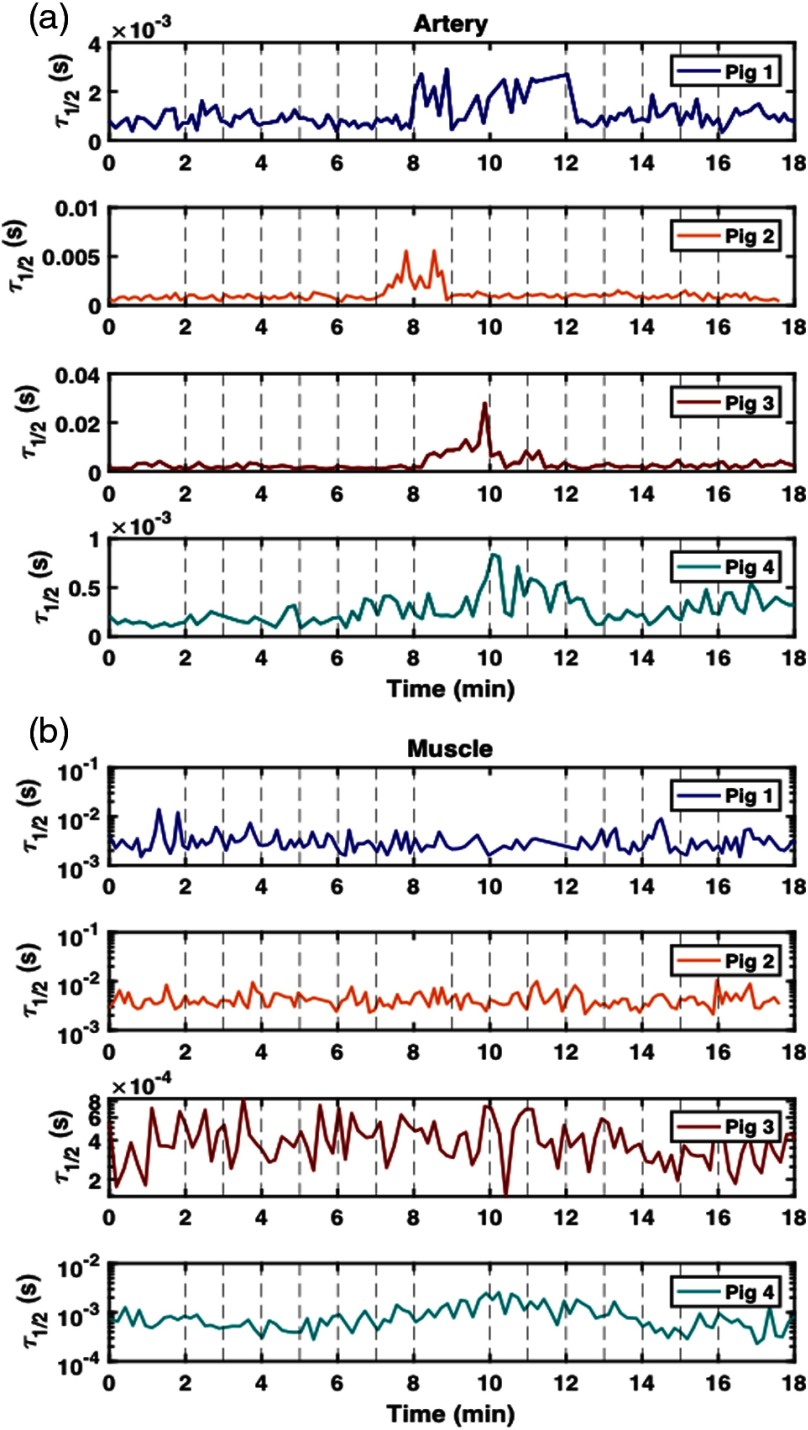
$\tau_{1/2}$ values calculated from autocorrelation curves collected at artery sites on pigs 1, 2, 3, and 4—occlusion studies were performed on all animals using a Hoffman clamp placed over the artery. Each study began with the Hoffman clamp fully opened (3.2 mm). Incremental tightening of the clamp (−0.4  mm) occurred every minute after the first 2 min, with full occlusion reached at 8 min for pigs 1, 3, and 4—for pig 2, full occlusion was reached after 7 min. For pig 1, the fully occluded state was maintained for 4 min, after which, incremental untightening (+0.4  mm) of the clamp occurred every minute until the clamp reached its original position (3.2 mm). For pigs 2, 3, and 4, the fully occluded state was only maintained for 2 min. (a) A probe was secured to the artery downstream from the Hoffman clamp via sutures [see Fig. 2(d)]. Only pigs 2 and 3 recorded a significant arterial response during the occlusion study. (b) A probe was secured to the muscle via sutures [see Fig. 2(c)]. Pigs 1 and 2 demonstrated considerably more noise at the muscle site than at any other tissue site. Pig 4 demonstrated the greatest response to the occlusion protocol; however, it was difficult to measure a response at the muscle site for all four animals.

Figure 7(a) shows the $\tau_{1/2}$ values calculated from the DCS autocorrelation curves collected from four animals at the artery site. As discussed in Sec. 2.2, the artery probe was placed downstream 1 to 1.5 cm away from the Hoffman clamp. The probe was secured via sutures tied around the artery (these sutures did not penetrate the tissue). The strongest recorded response to the occlusion protocol at the artery site was in pig 3. Figure 7(b) shows the $\tau_{1/2}$ values for data collected on the muscle sites on four distinct animals. Pig 4 was the only animal for which the device registered a response to the occlusion study at the muscle site.

**Fig. 8:**
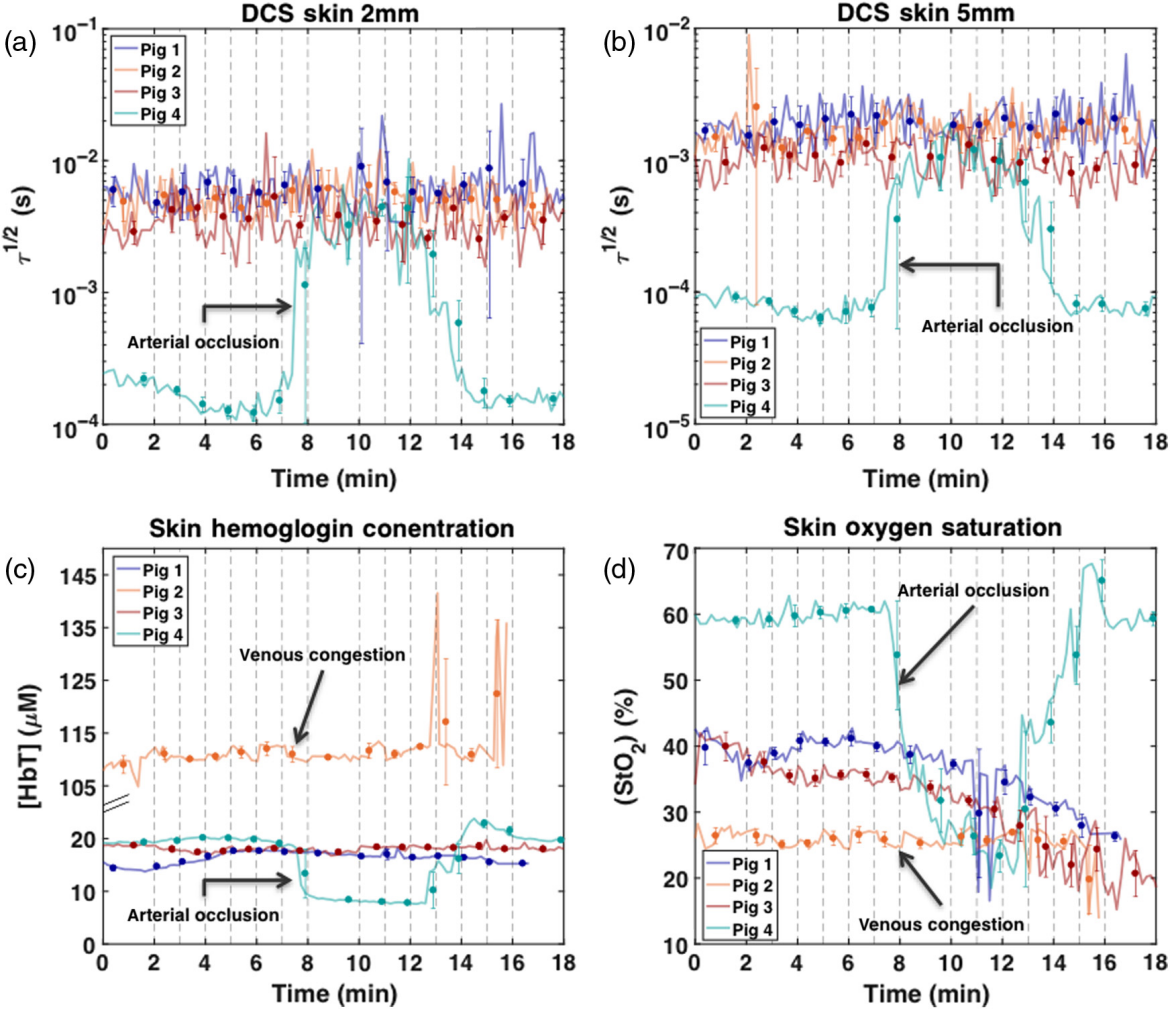
DCS and DRS skin results—occlusion studies were performed on all pigs using a Hoffman clamp placed over the artery. A skin patch was secured to the artery downstream from the Hoffman clamp via sutures [see Fig. 2(c)]. For pigs 3 and 4, the study began with the Hoffman clamp fully opened (3.2 mm). Incremental clamp tightening (−0.4  mm) occurred every minute after the first 2 min, with full occlusion reached at 8 min. After 10 min, incremental clamp untightening (+0.4  mm) occurred every minute until the clamp reached its original position (3.2 mm). (a) and (b) display DCS $\tau_{1/2}$ values calculated from autocorrelation curves measured at skin sites on pigs 1 to 4 using 2- and 5-mm source–detector separations, respectively. (c) and (d) display hemodynamic variables, total [HbT], and ${\mathrm{StO}}_{2}$ extracted from DRS spectra measured at skin sites on pigs 1 to 4. After DRS data acquisition on pig 2, the surgeon reported spontaneous venous congestion in the flap.

Figure 8 shows the results of DCS and DRS measurements taken at skin sites during occlusion studies on four pigs. As mentioned in Sec. 2, DCS measurements were taken simultaneously using a single probe with 2- and 5-mm source–detector separations for each animal (see Fig. 2). DRS measurements were taken exclusively on skin at sites adjacent to the DCS sites. The top two plots in Fig. 8 show the $\tau_{1/2}$ values calculated from the 2-mm (a) and 5-mm (b) DCS measurements. The bottom two plots show hemoglobin concentration (c) and percent tissue oxygen saturation (d). Notice the correlation between the DRS and DCS data for pig 4 as well as the relatively sharp transition between the occluded and open states as indicated by the open distance of the Hoffman clamp.

**Fig. 9:**
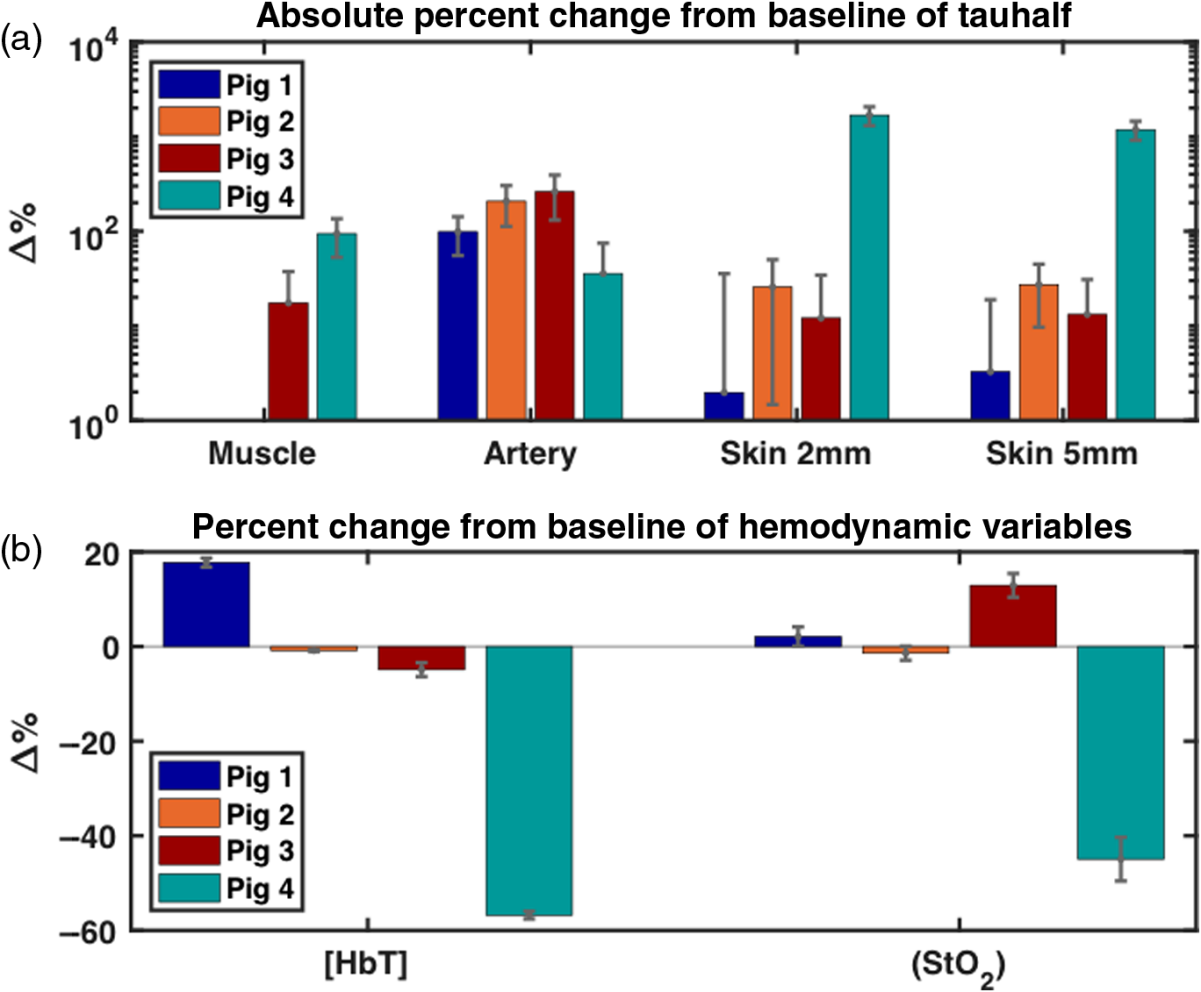
(a) Measured DCS response to occlusion at four distinct tissue sites (artery, muscle, skin 2 mm, and skin 5 mm) across four animals—the calculation of DCS system response is discussed in detail in Sec. 2.4.2. The large response in $\tau_{1/2}$ for pig 4 at the 2- and 5-mm skin sites necessitates the DCS results being plotted on a logarithmic scale. Because of this, negative percent changes from baseline are absent from Fig. 9(a) (pig 1: muscle and pig 2: muscle). In addition, any uncertainty whose range is larger than the size of its corresponding value does not display a lower bound error bar (pig 1: skin 2 mm, skin 5 mm; pig 3: muscle, skin 2 mm, skin 5 mm; and pig 4: artery). (b) Measured DRS system response to occlusion at skin sites across four animals—the calculation of system response is discussed in detail in Sec. 2.5.

Figure 9 summarizes the measured occlusion response in the reported experiments. Measured response was defined as the percent change from the baseline measurement at full occlusion. Baseline was defined as the points in the protocol where the Hoffman clamp was maximally opened. Measurement uncertainty was calculated using the propagation of error formula. A detailed explanation of how system response was calculated from DCS and DRS data can be found in Secs. 2.4.2 and 2.5, respectively.

As noted in the caption, the large response in $\tau_{1/2}$ for pig 4 at the 2- and 5-mm skin sites necessitates the DCS results being plotted on a logarithmic scale. Because of this, negative percent changes from baseline are absent from Fig. 8(a) (pigs 1 and 2, muscle). In addition, any uncertainty whose range is larger than the size of its corresponding value does not display a lower bound error bar (pig 1: skin 2 mm, skin 5 mm; pig 3: muscle, skin 2 mm, skin 5 mm, and pig 4: artery).

### 3.3 Anastomosis Studies

**Fig. 10:**
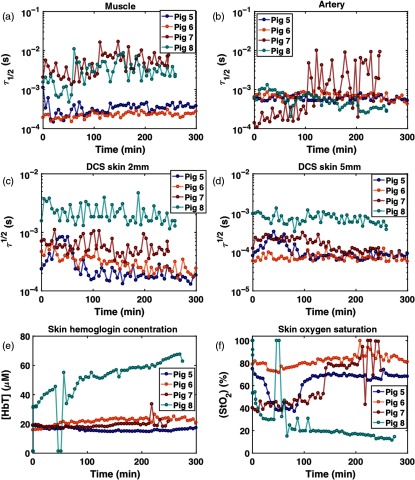
DCS and DRS results during 5-h monitoring. (a) $\tau_{1/2}$ in muscle, (b) $\tau_{1/2}$ in artery, (c) $\tau_{1/2}$ in skin 2 mm, (d) $\tau_{1/2}$ in skin 5 mm, (e) skin [HbT], and (f) skin ${\mathrm{StO}}_{2}$. The pig 8 results suggest that perfusion may have been compromised prior to the failure simulation.

In all four animals, the surgeons did not find any signs of flap failure during the 5-h monitoring after anastomosis. Thus, additional monitoring on facilitated venous and arterial failures followed. For the 5-h period, DRS and DCS data were acquired without any technical issues. Figure 10 shows the results of 5-h monitoring. In three of the four animals, the estimated $\tau_{1/2}$ in the muscle and $\tau_{1/2}$, ${\mathrm{StO}}_{2}$, and [HbT] in the skin sites during 5-h monitoring are relatively constant in time, and the time-averaged values are consistent among animals in a normal range, ${\mathrm{StO}}_{2}=68.4\pm 11.8\%$ (mean±std), [HbT]=19.0±2.9  μM, indicative of successful reperfusion via anastomosis. In one animal (pig 8), the data showed increasing [HbT] higher than 65  μM and decreasing ${\mathrm{StO}}_{2}$ below 15%, and higher $\tau_{1/2}$ by 1 order of magnitude than the other animals at the end of the monitoring. This could suggest venous congestion as observed in pig 2, but this was not noted by the surgeon using physical exams. It is also noted that ${\mathrm{StO}}_{2}$ in pig 5 decreased to ∼40% at 40-min postanastomosis and recovered to 70% after 1 h while [HbT] maintained a constant level. Within the same time window, $\tau_{1/2}$ at the 2- and 5-mm skin sites increased and then decreased to the baseline value. The lowered ${\mathrm{StO}}_{2}$ during this period may be attributed to a temporary decrease in blood flow, supplying insufficient oxygen to the tissues.

**Fig. 11:**
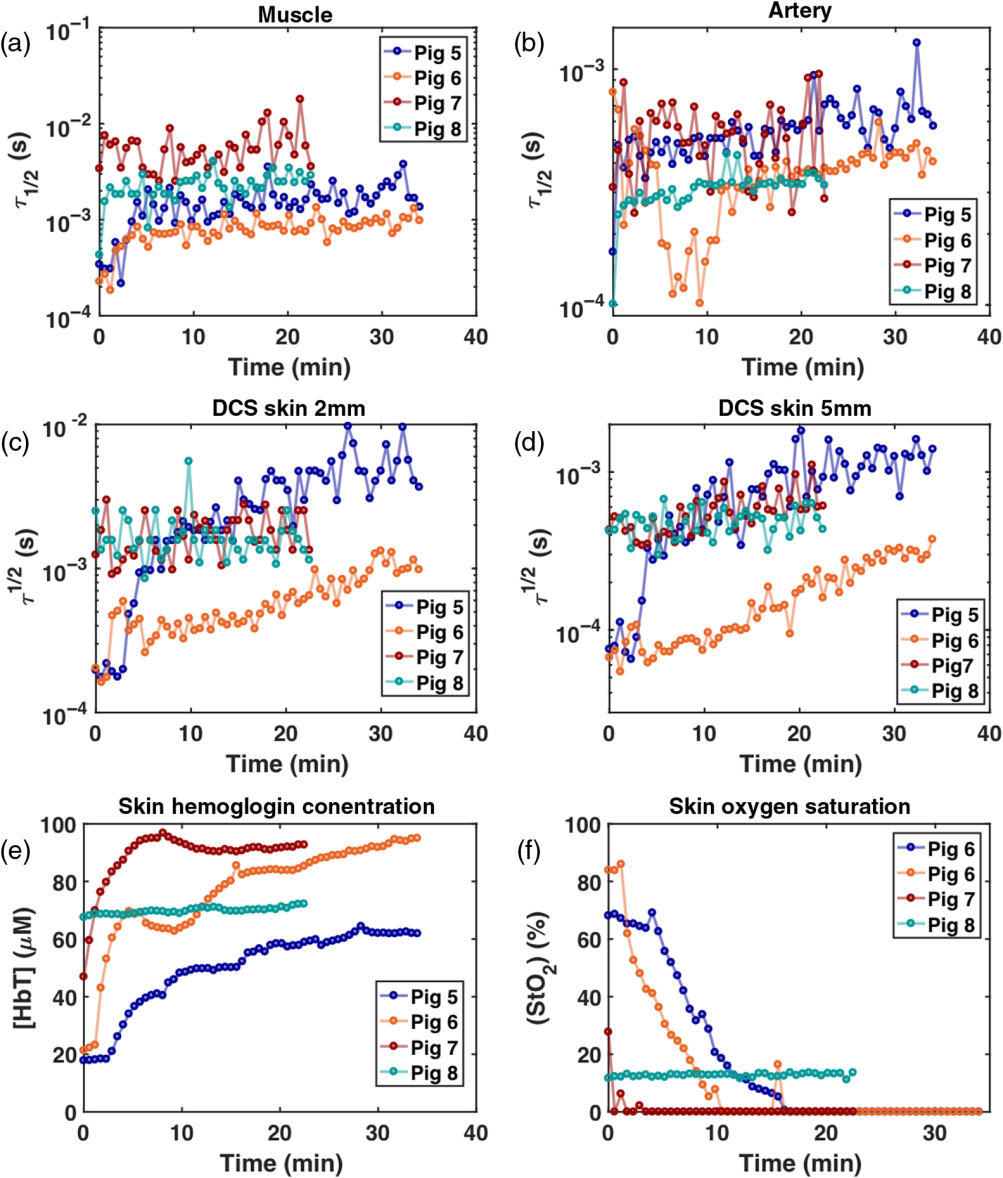
DCS and DRS results during venous failure. (a) $\tau_{1/2}$ in muscle, (b) $\tau_{1/2}$ in artery, (c) $\tau_{1/2}$ in skin 2 mm, (d) $\tau_{1/2}$ in skin 5 mm, (e) skin [HbT], and (f) skin ${\mathrm{StO}}_{2}$.

Figure 11 shows the DCS and DRS data during the venous failure simulation. In pigs 5 and 6, it is clearly observed that $\tau_{1/2}$ for the muscle and skin 2- and 5-mm sites, and [HbT] gradually increase while ${\mathrm{StO}}_{2}$ decreases. In pig 7, [HbT] increases as it does in pigs 5 and 6 while ${\mathrm{StO}}_{2}$ reaches almost zero percent with the increased $\tau_{1/2}$ in muscle compared to the baseline in Fig 10. The blood flow that becomes slower in a relatively faster time would deprive the tissue of oxygen more rapidly. The data from pig 8 are consistent with the values observed during 5-h monitoring.

**Fig. 12:**
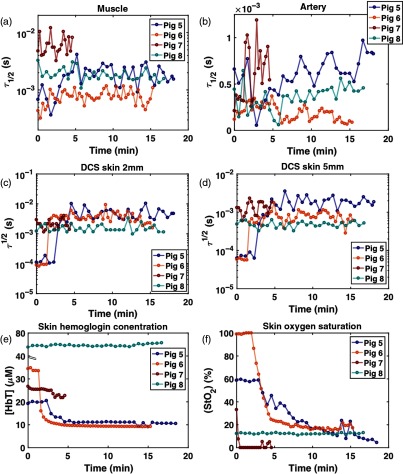
DCS and DRS results during artery failure. (a) $\tau_{1/2}$ in muscle, (b) $\tau_{1/2}$ in artery, (c) $\tau_{1/2}$ in skin 2 mm, (d) $\tau_{1/2}$ in skin 5 mm, (e) skin [HbT], and (f) skin ${\mathrm{StO}}_{2}$.

Figure 12 shows the DCS and DRS data during the arterial failure simulation. Like the venous failure, data from pigs 5 and 6 feature the most obvious changes in $\tau_{1/2}$, ${\mathrm{StO}}_{2}$, and [HbT]. Immediately after the artery is ligated, $\tau_{1/2}$ dramatically increases in muscle, skin 2 and 5 mm, and [HbT] and ${\mathrm{StO}}_{2}$ rapidly decrease. The pig 7 data show only 5-min acquisition due to time limitations during the experiment. All parameters in pig 8 are the same as those observed during 5-h monitoring and venous occlusion simulation.

**Fig. 13:**
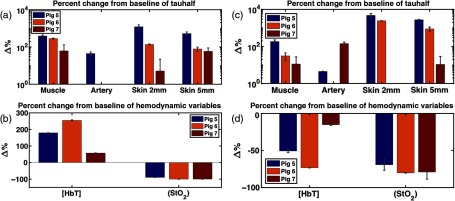
Relative changes of perfusion parameters after 15 min lapsed from baseline for (a and b) the simulated venous and (c and d) arterial compromise in pigs 5, 6, and 7. Baseline is computed as the average of the first three data points. Likewise, three acquisitions around 15 min (5 min for pig 7) are averaged for calculation of relative changes. The error bars were calculated using the propagation of error equation as a function of the mean and standard deviations of the baseline and 15-min data points. With the exception of artery and skin 2 mm for pig 7, the $\tau_{1/2}$ values increased for all sites and animals, indicating slower blood flow. ${\mathrm{StO}}_{2}$ also decreases in both compromises, and only [HbT] shows an opposite direction of change, which could be a key parameter for distinguishing between venous and arterial compromise in the flap.

Figure 13 summarizes the percent relative changes of each parameter in pigs 5 to 7 during occlusion simulation, from the baseline to the 15-min (5 min for pig 7) time point in response to the simulated venous (a and b) and arterial congestion (c and d). The baseline is defined as an average of first three data points. The large response in $\tau_{1/2}$ for pigs 5 and 6 necessitates the DCS results being plotted on a logarithmic scale. Because of this, negative percent changes from baseline are absent from Fig. 13(a) (pigs 6 and 7, artery) and Fig. 13(c) (pig 6, artery and pig 7, skin 2 mm) (pigs 1 and 2, muscle). In addition, any DCS uncertainty whose range is larger than the size of its corresponding value does not display a lower bound error bar [Fig. 13(a): pig 7: muscle, skin 2 mm; Fig. 13(c): pig 7: artery, skin 5 mm].

In both compromises, $\tau_{1/2}$ at muscle and skin 2 and 5 mm in all three animals increase greatly from baseline. Particularly in pigs 5 and 6, the $\tau_{1/2}$ at muscle, skin 2 mm and 5 mm increase by 1 to 2 orders of magnitude. ${\mathrm{StO}}_{2}$ decreases by 96.3±6.3% and 76±6.1% in venous and artery congestion, respectively. While [HbT] increases by 163.3±99% in venous failure, it decreases by 46.3±29.4% in arterial failure. [HbT] is the only parameter exhibiting an opposite trend between two compromises, as demonstrated in the other commercial oximetry systems.^36^ These plots reveal the potential of our multimodal diffuse optics device to distinguish between venous and arterial compromises based on the direction of changes in [HbT].

## 4 Discussion

This study aimed to test a compact, diffuse optical system based on DRS and DCS that quantified the changes of tissue perfusion parameters (blood flow—$\tau_{1/2}$, tissue oxygenation—${\mathrm{StO}}_{2}$, and total hemoglobin concentration—[HbT]) during different degrees of arterial occlusion and different simulated compromises following 5-h postoperative monitoring on an *in vivo* porcine flap model. To demonstrate the instrument’s multisite and multichannel monitoring capabilities, three different sites (skin, muscle, and artery) were monitored simultaneously via custom optical probes on a total of four animals.

For the arterial occlusion study, several experimental challenges were noted, including inconsistency in changes of perfusion parameters upon occlusion across different sites and different animals. For the $\tau_{1/2}$ measurements [Fig. 8(a)], the strongest response was measured on the skin site of pig 4 (both 2- and 5-mm source–detector separation). The next two strongest responses occurred at the artery site for pigs 3 and 2, respectively. The smallest response, which was still visible by eye in the $\tau_{1/2}$ plots, occurred for pig 4 at the muscle site. From the DRS results, both [HbT] and ${\mathrm{StO}}_{2}$ displayed the strongest response at the skin site of pig 4. Pig 4 was the only animal for which a visible change in spectra was observed during the occlusion study. In general, the DCS and DRS results from the skin site yielded congruent results for pigs 3 and 4. Because DCS and DRS were measured during separate occlusion studies for pigs 1 and 2, the $\tau_{1/2}$ results for these animals are not directly comparable to the [HbT] and ${\mathrm{StO}}_{2}$ results.

The changes of $\tau_{1/2}$, ${\mathrm{StO}}_{2}$, and [HbT] shown in the skin site of pig 4 at full occlusion demonstrate that this optical system is potentially capable of identifying an arterial compromise in tissue flaps. The observed increase of $\tau_{1/2}$ (slow blood flow) and decrease of ${\mathrm{StO}}_{2}$ and [HbT] were consistent with our physiological prediction. Interestingly, during the DRS occlusion study for pig 2, the surgical team noted color changes in the elevated flap consistent with venous congestion—specifically, the flap began to have a purple hue. The DRS analysis of pig 2 yielded results in support of this observation. Specifically, high [HbT] and low ${\mathrm{StO}}_{2}$ were measured [Figs. 6(c) and 6(d)], suggesting that blood was pooling and unable to drain. These changes are consistent with a previous clinical study using commercial LDF + spectrophotometry system^37^ and the results of our simulated venous failure experiment (Fig. 11).

The inconsistent responses to occlusion observed between the animals and sites could result from a combination of variation between animals, the operator learning curve, and probe-tissue coupling issues. The results for pig 4 reveal that $\tau_{1/2}$, ${\mathrm{StO}}_{2}$, and [HbT] change over a small range of occlusion as indicated by the distance of the opening in the Hoffman clamp. The small responses seen in pigs 1 to 3 may partially be attributed to measurement process refinement; however, we cannot rule out the possibility of sample-to-sample variation. For example, artery size variation across animals may also explain the differences observed when different animals responded to occlusion [this is most notable in Fig. 6(a)]. Experience gained during this four-pig study could also explain the relative quality of the data from the last pig studied (pig 4) and the following long-term monitoring study (pigs 5 to 8). In addition, the surgeons reported difficulty in fixing the artery probe such that it provided an adequate signal. The complexity of securing several fiber-coupled patches—while maintaining a consistent clamping protocol in the surgical suite—presented several opportunities for systemic error to affect results.

An interesting observation made over the course of the arterial occlusion study involved the almost binary response of the device to the occlusion protocol, in spite of linearly incremental distance changes. We are confident that the protocol stages highlighted in this paper represented no occlusion (at 0 to 2 min and at 16 to 18 min) and full occlusion (at 8 to 10 min); we directly correlated 0% occlusion to the fully opened or reopened Hoffman clamp distance and 100% occlusion to the fully closed Hoffman clamp distance. However, it was difficult to estimate the degree of occlusion at intermediate distances. Both DRS and DCS data from skin in pig 4 changed dramatically only at the nearly full occlusion distance (0.4 to 0.8 mm). After their initial change, these parameters remained stable until a period after the clamp was released to 1.5 mm at around 13 min [see Figs. 3(d) and 7]. This nonlinear response could be attributed to hysteresis of the arteries’ elasticity and to the unidirectional closing of the clamp. The artery’s deformation upon a unidirectional force could still allow for blood to flow, even at a very small clamping distance. Once the artery is completely occluded, a greater distance (1.5 mm) is required for the artery to extend again, in comparison with the distance necessary to initiate full occlusion (0.4 to 0.8 mm). Perhaps, a different occlusion mechanism, such as a balloon cuff^6^ that can press vessels from all directions, would provide more precise and predictable partial occlusion.

The anastomosis studies on four additional animals demonstrate our device’s technical feasibility to continuously monitor postoperative perfusion in a free microvascular flap. The first couple of hours after surgery are a critical time window for flap salvage, and continuous monitoring would improve the salvage rate by instantly alerting the clinical team about abnormal perfusion. Moreover, the results of 5-h-long monitoring after successful anastomosis reveal that absolute quantification of perfusion via our diffuse optics techniques would make it possible to establish a threshold, because the three perfusion parameters ($\tau_{1/2}$, ${\mathrm{StO}}_{2}$, and [HbT]) measured on three animals (pigs 5 to 7) are consistent during 5-h monitoring and deviate largely from their baseline during the simulated compromises.

The monitoring results in the simulated failures confirm the observations made for the first four animals and lend further credence to the device’s potential in detecting spontaneous flap failure and distinguishing between arterial and venous compromise. The physiological mechanism behind the recorded changes of each parameter responding to two different compromises is straightforward. Ligation of the artery inhibits oxygenated blood from supplying the flap, resulting in an immediate drop in blood flow, [HbT], and ${\mathrm{StO}}_{2}$. Occluding the vein causes a gradual decrease in flow speed and the accumulation of deoxyhemoglobin inside the tissue, leading to a gradual increase of [HbT] and decrease of ${\mathrm{StO}}_{2}$. Since there is still an arterial inflow to the flap with a blocked venous outflow, perfusion parameters represent a prolonged response. Measurements of either tissue oxygen saturation or blood flow alone can accurately identify hypoxia in tissues but not the cause of hypoxia.^38^

The result of pig 8 is controversial. The acquired data during 5-h postanastomosis (Fig. 10) represent a similar response with both spontaneous (pig 2 in Fig. 8) and artificial venous congestions (pigs 5, 6, and 7 in Fig. 11). However, the surgeons did not identify any sign of this during their physical exams. Although we cannot exclude the possibility of device error, it could be interpreted that our device is capable of detecting a subtle change in perfusion, which might not be caught by early physical signs.

For the DCS data analysis in our study, a heuristic parameter, $\tau_{1/2}$, was selected to relate the data to blood flow. This was chosen in place of the estimation of blood flow index (BFI), usually computed using the DE. The experiments presented in this study were designed to alter the optical properties (mostly absorption coefficients) of microvascular-free flaps. This choice to use $\tau_{1/2}$ was made because the DE typically assumes constant optical properties when estimating BFI, which could lead to significant errors in analysis.^39^ Another minor issue is the possible breakdown of the DE in a small source–detection separation,^40^ such as our pedicle and skin DCS probe. Since the device is capable of estimating the optical properties of the sample up to 650 nm, our next step will be to extrapolate these to estimate the optical properties at the DCS wavelength, 785 nm. After thorough investigation on validity of the DE for our probe geometry, BFI evaluation would be helpful to establish an objective threshold for flap failure in the future in comparison with other flow assessment techniques currently used in clinics. For a more accurate BFI estimation with varying optical properties, colocalized and simultaneous optical measurements between DCS and DRS may be required; this can be achieved through an optical probe design combining source and detection fibers of DCS and DRS. Other experimental setup refinements—such as improvements in probe fixations, the addition of partial venous occlusion, and a larger sample size—will enable more rigorous device testing.

## 5 Conclusion

In conclusion, a compact multimodal diffuse spectroscopy system was tested on an *in vivo* porcine flap model. Three major perfusion-related parameters ($\tau_{1/2}$, ${\mathrm{StO}}_{2}$, and [HbT]) were monitored during the arterial occlusion, postoperative phase, and the facilitated vascular compromises on multiple flap tissue sites. The monitoring results suggest promise for this technology to be used for long-term monitoring of free microvascular flaps. The compact construction of the device along with its noninvasive and quantitative nature and ability to detect arterial and venous compromise would make this technology suitable for clinical translation to compete with current clinical devices.
